# Real-World Evidence of User Engagement With Mobile Health for Diabetes Management: Longitudinal Observational Study

**DOI:** 10.2196/22212

**Published:** 2020-11-06

**Authors:** Anna-Katharina Böhm, Morten Lind Jensen, Mads Reinholdt Sørensen, Tom Stargardt

**Affiliations:** 1 Hamburg Center for Health Economics University of Hamburg Hamburg Germany; 2 Novo Nordisk A/S Søborg Denmark

**Keywords:** user engagement, user activity, mHealth, diabetes mellitus, diabetes apps

## Abstract

**Background:**

Patient support apps have risen in popularity and provide novel opportunities for self-management of diabetes. Such apps offer patients to play an active role in monitoring their condition, thereby increasing their own treatment responsibility. Although many health apps require active user engagement to be effective, there is little evidence exploring engagement with mobile health (mHealth).

**Objective:**

This study aims to analyze the extent to which users engage with mHealth for diabetes and identify patient characteristics that are associated with engagement.

**Methods:**

The analysis is based on real-world data obtained by Novo Nordisk’s Cornerstones4Care Powered by Glooko diabetes support app. User engagement was assessed as the number of active days and using measures expressing the persistence, longevity, and regularity of interaction within the first 180 days of use. Beta regressions were estimated to assess the associations between user characteristics and engagement outcomes for each module of the app.

**Results:**

A total of 9051 individuals initiated use after registration and could be observed for 180 days. Among these, 55.39% (5013/9051) used the app for one specific purpose. The average user activity ratio varied from 0.05 (medication and food) to 0.55 (continuous glucose monitoring), depending on the module of the app. Average user engagement was lower if modules required manual data entries, although the initial uptake was higher for these modules. Regression analyses further revealed that although more women used the app (2075/3649, 56.86%), they engaged significantly less with it. Older people and users who were recently diagnosed tended to use the app more actively.

**Conclusions:**

Strategies to increase or sustain the use of apps and availability of health data may target the mode of data collection and content design and should take into account privacy concerns of the users at the same time. Users’ engagement was determined by various user characteristics, indicating that particular patient groups should be targeted or assisted when integrating apps into the self-management of their disease.

## Introduction

### Background and Aims

Diabetes causes elevated blood glucose (BG) because the body is not able to produce any or sufficient amounts of insulin or is not capable of processing it effectively. Patients have an increased risk of developing life-threatening complications caused by prolonged elevated glucose levels [1]. Although for people with early stage type 2 diabetes (T2D), a healthy diet, exercise, and weight loss may be sufficient to maintain near normal BG, people with type 1 diabetes (T1D) or progressed T2D require insulin therapy and more intensive glucose monitoring [2]. Thus, if treated adequately, complications can be avoided or delayed, but this requires thorough self-care and adherence to treatment [3].

The ongoing digital revolution combined with the increasing prevalence of diabetes has led to an abundance of mobile health (mHealth) apps promising novel opportunities for self-monitoring and treatment guidance for diabetes patients [4]. Functions such as providing health information, medication reminders, remote monitoring, and mobile analytics may increase the users’ health literacy, support patients to play a more active role in managing their disease, and promote adherence to treatment [4,5]. In addition, mHealth may enable sharing of data between patients and health care professionals (HCPs), which could support an improved patient-HCP dialogue [6]. Thus, if utilized optimally, mHealth may be an opportunity to increase quality of care while at the same time offering the potential to reduce costs for health care systems [7-9].

On the other hand, factors such as low digital competence or limited availability of technology could reduce the use and cost-effectiveness of mHealth. In addition, data privacy can be a concern for patients and may lead users to omit use or enter artificial information [10,11]. Furthermore, it is inherently difficult for patients and HCPs to select the most appropriate app for the individual patient, as there are over 300,000 health apps with different key features available on the market [12].

Although the variety and popularity of mHealth for diabetes patients is increasing, there is limited evidence about who uses the apps, the users’ engagement, and the apps’ effectiveness [11]. Surveys from the United States and Germany reveal that users of health apps were younger individuals; more often female; had an advanced education, higher income, and better health literacy [13-16]. However, these studies analyzed health apps in general and ignored disease-specific characteristics of use. Similarly, studies exploring user engagement often focused on other disease areas than diabetes [17-20]. A systematic review analyzing the pattern of user engagement of technology-based interventions for T2D patients emphasized the need for studies reporting on engagement, examining associations between user characteristics and engagement, and standardizing how user engagement is reported [21].

### Objectives

This study explores real-world data obtained by Novo Nordisk’s Cornerstones4Care (C4C) patient support app (the app) for diabetes. The aim of this study was to study the use of mHealth among diabetes patients, here represented by the users of the app. In particular, the study aims to analyze how intensively users engage with the app and to identify patient characteristics that are associated with engagement with the app. Addressing these aims will help to understand how mHealth apps can be used effectively and what areas could be optimized.

## Methods

### General Overview of the App

C4C is a US patient support program for diabetes patients, funded by Novo Nordisk A/S. It includes the option to use the *Cornerstones4Care Powered by Glooko* mHealth app. The app has been available since June 2017, and approximately 30,000 users have installed the app by October 2019 and provided consent to use the data for research purposes. Installation does not require prescription by an HCP. The patient support program and the app provide information materials and tools to monitor nutrition, fitness, medication, and glucose levels (the modules). The app allows users to synchronize data from BG meters, continuous glucose monitoring (CGM) devices, insulin pumps, and external fitness and health devices. In addition, the user can view historic data and trend graphs, set reminders for testing their BG or taking medication, and add custom notes. Thus, it brings together different diabetes data relevant for effective disease management. In addition to this, the app allows sharing of data between the patient and the HCP.

### Study Outcomes

A major challenge when assessing how users engage with mHealth is the lack of consensus on how to assess user engagement [22]. Therefore, we based our choice of engagement metrics on a theoretical framework defining user engagement of technology as a progress comprising 4 distinct stages: point of engagement, period of sustained engagement, disengagement, and reengagement [23]. We established 6 metrics capturing different aspects of user engagement. Table 1 summarizes the application of the conceptual framework in our study.

To account for censoring, that is, shorter observation periods for users who have downloaded the app more recently, we restricted our sample to users who had started using the app before April 26, 2019, and assessed user activity during the first 180 days after initiation.

**Table 1:** Conceptual framework underlying user engagement metrics based on the study by O’Brien and Toms, 2013.

Conceptual framework and metric names		Definition	
**Point of engagement**			
	Activity delay		Days from consent date to first active day
**Period of engagement**			
	User activity ratio		Number of active days divided by number of potentially active days (here 180)
	Longevity		Days from first until last active day (maximum=180)
	Recency		Average number of days between entries
	Regularity		Coefficient of variation of number of days between entries
**Discontinuation**			
	Persistence		Days until 28 days discontinuation
	Dropouts		Illustrated in Figure 1
**Reengagement**			
	Engagement pattern		Illustrated in Figure 2

The core metric is the user activity ratio (UAR). It counts the number of active days (ie, days with at least one entry) of user *i* in module *m*, divided by the number of potentially active days (here 180 days):


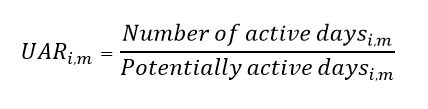


In addition to this, we investigated the *activity delay*, which reflects the number of days between the consent date and first active use. Moreover, as proposed by Rahman et al [18], *longevity* captures the time from the initial use until the final entry (maximum 180 days). *Persistence* was expressed as the number of days until the first discontinuation of use within 180 days, where discontinuation was defined as a gap of >28 concurrent inactive days. The size of the permissible gap was based on the 0.9 percentile of all average user gaps. If users did not discontinue use, persistence was set to 180. In accordance with Taki et al [20] and Peterson and Carrabis [24], we also assessed the *recency*, which was defined as the average time between active days for each user. To quantify the *regularity* of activity, we additionally calculated the coefficient of variation of the time between active days, that is, the standard deviation of the time between active days divided by the recency. This number allows a direct comparison between the modules.

Finally, we calculated alternative versions of the UAR with the number of potentially active days as the (1) longevity or (2) persistence.

In addition to engagement, the metrics reflect the period and amount of data collection and, therefore, the availability of rich health information. Kendall correlations for nonlinear data were calculated to assess the redundancy of the metrics. All metrics were calculated for each of the 5 modules and for the overall app.

### C4C Data

The C4C data set includes all app entries (manual and automated) collected during the period from app launch (June 28, 2017) to October 21, 2019. The data set is structured according to the 5 main modules of the app, namely, *food intake*, *exercise*, *medication intake*, *BG*, and *CGM*. In addition, it contains basic user information. The app contains no personally identifiable data, and users have provided their consent for data sharing. The data were aggregated for analysis.

#### User Information

Basic user information covers consent information (eg, date of consent), sociodemographic data (age and gender), diabetes type, bodyweight measures (latest height and weight), and a medication profile (medication type, medication name, and time at which the user indicated treatment initiation). Self-reported age is the only mandatory information to be shared when using the app. If information on height and weight was available, we calculated the user’s BMI.

On the basis of the medication profile, we further extracted if a user had registered insulin (yes=1 and no=0), other injectables (yes=1 and no=0), orals (yes=1 and no=0), the total number of registered medications, and the exact number of oral substances. On the basis of the medication name, we also extracted if a user registered fast-acting bolus insulin (yes=1 and no=0) because they represent patients with T1D and progressed T2D, thus patients who may require more intensive monitoring. The resulting treatment regimen information served as a proxy for disease severity.

Furthermore, we combined the information on diabetes type and medication profile to determine if a user was newly diagnosed. T1D patients were considered as recently diagnosed if treatment with insulin started within 180 days before the consent date. T2D patients were considered as recently diagnosed if (1) medication was limited to metformin, which is the typical first-line treatment for people with T2D, and (2) treatment started within 180 days before the date of consent.

#### Food Intake

Data on food intake include information on registered carbs, fat, protein, and calories. The user can either enter the meal manually, select a meal from a list, or scan the barcode of groceries. The data set stores the time the user indicated that the food was consumed and a timestamp marking the data entry.

#### Exercise

Exercise data provide a daily summary of the user’s activity, including the duration, distance, burned calories, steps, floors, and elevation. The user can either enter an activity manually, pick one from a list, or synchronize with an external health app (as the app must be opened to sync, our engagement metrics can serve as a proxy for user activity in [partly] automated modules). The data set covers the day of the daily summary of the user’s activity as well as a timestamp of the data record, that is, the day of manual entry by the user or synchronization from an external app.

#### Medication Intake

Data about medication intake contain information on the registered medication, including the name, type (oral, insulin, and other injectables), and dosage. The user can pick the medication from a predefined list or make a manual entry. The data cover the time at which the user indicated the medication was taken and a timestamp marking the data entry by the user.

#### BG Values

The app allows the storage of BG values (mg/dL) obtained by BG meters. A BG meter is a medical device used to determine the concentration of glucose in the blood. It detects the level of sugar in capillary blood taken from a finger prick. The data set contains information on the time of BG measurement and a data entry timestamp (manual entry by the user or synchronized from a BG device via Bluetooth; this function is only available in few BG meters). In addition, users can add tags indicating if a reading occurs during fasting or is taken postprandially. Meal tags can also be synched from the BG meter. If available, we used this information to assess if the user’s baseline BG, that is, their first preprandial glucose measurement or their first value after nighttime (5:00 AM until 9:00 AM), was well controlled (yes=1 and no=0). The threshold of 80-130 mg/dL was adopted from official recommendation by the American Diabetes Association (ADA) [25].

#### CGM Data

In contrast to BG meters, which only capture BG at a certain point in time when a user performs the relevant actions, CGM devices typically generate 5-min interval data. An electrode is placed under the skin, while the transmitter sends data to a separate receiver, and the data are then sent to the app via Bluetooth. The data set covers the glucose values (mg/dL) as well as a trend arrow representing the visual change in the glucose trend. It contains information on the measurement time and a timestamp of the phone transmission (similar to the exercise module, the engagement metrics can serve as a proxy for user activity in [partly] automated modules because the app needs to be opened to sync). In this study, baseline glycemic control was assessed based on recommended target ranges established by an international expert panel in 2019 [26]. We determined that users were in good glycemic control if they exceeded the recommended 70% of time in range (TIR; 70-180 mg/dL) within the first 14 days of observations in which sufficient data were available.

### Statistical Analysis

#### Exploratory Data Analysis

Descriptive statistics of user characteristics and study outcomes are presented as means, standard deviations, and ranges for continuous variables and as counts and percentages for categorical variables. If the values of self-reported data were implausible, observations were treated as unknown.

Uptake and discontinuation of use for each module are visualized as graphs. Survival curves were created to show the proportion of users who were still active (on that day or on a later day) over time. Event diagrams, moreover, visually summarize the pattern of user activity.

#### Regression Analysis

Multivariate regression models were used to examine the associations between user characteristics and engagement (UAR) for each module. Specifically, we estimated general linear models with a beta distribution (for each module, the link function was chosen based on the smallest Akaike information criterion and Bayesian information criterion for model selection). By doing so, we account for the fact that engagement is double bounded by the values zero and one (100% user activity). If a user’s UAR was equal to 1, it was set to 0.99. Missing data, which is a major concern in self-reported data sets, were treated as a separate category (unknown) for all variables to retain the full number of observations. The equation of interest can be described as follows:


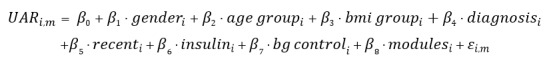


where *gender* represents the sex of the users, *age group* is a factor variable grouping users according to their age, *bmi group* represents the user’s BMI according to the official classification by the World Health Organization (WHO), *diagnosis* reflects the user’s diabetes type, *recent* is a proxy indicating if a user was recently diagnosed with diabetes, *insulin* is an indicator specifying if a user is treated with insulin (and therefore more severely ill), *bg control* indicates if the user’s fasting BG at baseline is well controlled, and *modules* reflects the total number of actively used modules per user. For a meaningful interpretation, the margins were calculated for each parameter. *P* values less than .10 were considered statistically significant in our analysis.

#### Sensitivity Analysis

Given that there is no consensus on what constitutes an adequate amount of user engagement, the calculation of activity metrics was repeated based on active weeks rather than active days to confirm the robustness of our findings. In addition, we confirmed the robustness of our findings by applying gamma and logged ordinary least squares regression as alternative regression specifications.

## Results

### Descriptive Results

#### User Characteristics

Among the 29,643 users who gave their consent after download, 12,685 initiated usage of the app. Table 2 summarizes user characteristics for the 9051 users meeting our inclusion criteria (ie, they initiated use and could be observed for 180 days); 63.50% (5747/9051) of the users reported being diagnosed with T2D and 13.48% (1220/9051) with T1D. Among users who shared information about their gender, 56.86% (2075/3649) reported to be female. It should be noted that only 40.32% (3649/9051) of all users provided information on their gender. More than half of the users were aged 50 years or older, and the average age of users was 50.4 years. The average BMI was 34.8, that is, in the obesity range according to the official classification by the WHO. However, the number was based on a subset of 36.69% (3321/9051) of users who shared information about their height and weight.

A total of 76.28% (6904/9051) of users set up a medication profile, with an average of 1.9 registered medications. Among these, 63.33% (4372/6904) registered oral medications and 56.34% (3890/6904) registered insulin.

Within users sharing information about their BG, a subset of 90.73% (4550/5015) provided sufficient data to assess their fasting BG, resulting in 34.48% (1569/4550) of well controlled users. Similarly, 86.76% (485/559) of CGM users provided sufficient data to assess their TIR. Among these, 38.14% (185/485) satisfied the recommended 70% of TIR, indicating good glycemic control.

**Table 2:** Descriptive statistics of user characteristics (N=9051).

Characteristics			Participants	Minimum value of user characteristics	Maximum value of user characteristics	Total unknown^a^
**Sociodemographics**						
	**Gender, n (%)**					5402
		Female	2075 (56.86)	0	1	
		Male	1574 (43.14)	0	1	
	**Age^b^ (n=8880), mean (SD)**		50.39 (13.48)	16	94	171
		<30 years, n (%)	719 (8.10)	0	1	
		30-40 years, n (%)	1184 (13.33)	0	1	
		40-50 years, n (%)	2103 (23.68)	0	1	
		50-60 years, n (%)	2541 (28.61)	0	1	
		60-70 years, n (%)	1737 (19.56)	0	1	
		>70 years, n (%)	596 (6.71)	0	1	
**Body measures**						
	Height^c^ (n=3646), mean (SD)		169.70 (10.57)	142.20	205.70	5405
	Weight^d^ (n=3507), mean (SD)		100.38 (29.24)	45.36	300.00	5544
	**BMI^e^ (n=3321), mean (SD)**		34.83 (9.79)	15.82	106.83	5730
		Underweight, n (%)	21 (0.63)	0	1	
		Normal weight, n (%)	354 (10.66)	0	1	
		Overweight, n (%)	716 (21.56)	0	1	
		Obese I, n (%)	851 (25.62)	0	1	
		Obese II, n (%)	612 (18.43)	0	1	
		Obese III, n (%)	767 (23.10)	0	1	
**Diagnosis**						
	**Type, n (%)**					894
		Type 1 diabetes	1220 (14.96)	0	1	
		Type 2 diabetes	5747 (70.45)	0	1	
		Prediabetes	715 (8.77)	0	1	
		Other	475 (5.82)	0	1	
	**Duration, n (%)**					2562
		Newly diagnosed	1780 (27.43)	0	1	
**Treatment regimen**						2147
	Number of registered medications (≥1) (n=6904), mean (SD)		1.86 (0.97)	1	11	
	Registered insulin, n (%)		3890 (56.34)	0	1	
	Registered bolus insulin, n (%)		2334 (33.81)	0	1	
	Registered other injectables, n (%)		1160 (16.80)	0	1	
	Registered orals, n (%)		4372 (63.33)	0	1	
	Number of oral substances (≥1) (n=4372), mean (SD)		1.38 (0.64)	1	6	
**Control of disease, n (%) of well-controlled users**						
	On the basis of baseline BG^f^		1569 (34.48)	0	1	4501
	On the basis of baseline CGM^g^		185 (38.14)	0	1	8566
**Demanded app modules, n (%)**						0
	Ever used medication tracker		3933 (43.45)	0	1	
	Ever used exercise		1579 (17.45)	0	1	
	Ever used food		3821 (42.22)	0	1	
	Ever used BG		5015 (55.41)	0	1	
	Ever used CGM		559 (6.18)	0	1	
**User scope**						0
	Number of modules used (>0) (n=9051) mean (SD)		1.65 (0.83)	0	5	
	Used 1 module, n (%)		5013 (55.39)	0	1	
	Used 2 modules, n (%)		2465 (27.23)	0	1	
	Used 3 modules, n (%)		1349 (14.90)	0	1	
	Used 4 modules, n (%)		203 (2.24)	0	1	
	Used 5 modules, n (%)		21 (0.23)	0	1	

^a^Total unknown reflects the number of users who did not share their information for each user characteristic.

^b^Age in years (between 16 and 100 years).

^c^Height in cm (between 140 and 220 cm).

^d^Weight in kg (between 45 and 300 kg).

^e^BMI groups according to the definition of the World Health Organization: underweight=BMI<18.5, normal weight=25>BMI≥18.5, overweight=30>BMI≥25, obese I=35>BMI≥30, obese II=40>BMI≥35, obese III=BMI≥40.

^f^BG: blood glucose.

^g^CGM: continuous glucose monitoring.

#### User Engagement

In addition to the user characteristics, Table 2 summarizes the use of the different modules of the app. Most initiated use of the BG module (5015/9051, 55.41%), medication module (3933/9051, 43.45%), and food module (3821/9051, 42.22%). A total of 17.45% (1579/9051) of all users applied the exercise module and 6.18% (559/9051) used the CGM module. It is noteworthy that more than 50% (5013 of 9051) of the individuals used the app for one single purpose, whereas only a total of 21 users took advantage of the entire app.

Table 3 presents the means and standard deviations of our study outcomes, that is, the 6 different user engagement metrics. Although most initiated use of the BG, medication, and food modules, the UAR was the lowest among the 3 modules (BG 0.07, medication 0.05, and food 0.05). In contrast, the UAR was highest for CGM (0.55) and exercise (0.37), both modules whose initial uptake remained rather low. Accordingly, activity delay was shortest for the medication and food modules (15 and 13 days, respectively), but low persistence (15 and 13 days, respectively) and longevity (23 and 20 days, respectively) showed that a high share of users stopped their engagement with the modules soon after initiation. In contrast, activity delay was longest for the CGM module (83 days), but once initiated, users remained active for an average of 133 days. A similarly high longevity (114 days) was observed for the exercise module. The recency of use was lowest for the exercise module (6 days) and highest for the BG module (18 days). Finally, it should be noted that the UAR turns from lowest to highest for the food module and for the medication module, if the denominator was defined as longevity or persistence, that is, if only the users’ active periods were considered.

**Table 3:** Descriptive statistics of user engagement metrics for each module.

Activity metrics		Medication (n=3933), mean (SD)	Food (n=3821), mean (SD)	Exercise (n=1579), mean (SD)	BG^a^ readings (n=5015), mean (SD)			CGM^b^ (n=559), mean (SD)		Any (N=9051), mean (SD)
**User activity ratio**										
	Denominator specified as 180 days	0.05 (0.15)	0.05 (0.14)	0.37 (0.35)	0.07 (0.15)			0.55 (0.41)		0.14 (0.27)
	Denominator specified as the longevity	0.79 (0.33)	0.82 (0.31)	0.61 (0.32)	0.51 (0.41)			0.72 (0.34)		0.65 (0.39)
	Denominator specified as the persistence	0.84 (0.28)	0.86 (0.26)	0.66 (0.29)	0.64 (0.37)			0.83 (0.26)		0.73 (0.33)
Activity delay		14.98 (60.06)	12.73 (54.33)	19.22 (78.10)		20.47 (64.77)	82.55 (149.70)		14.14 (58.96)	
Longevity		23.25 (46.95)	19.68 (42.56)	113.7 (78.50)	57.97 (65.32)			133.00 (66.50)		58.56 (71.90)
Persistence		15.20 (36.71)	13.14 (32.45)	100.30 (81.85)	31.11 (49.75)			105.60 (77.49)		40.00 (63.49)
Recency		9.06(18.83)	6.83 (16.76)	5.53 (11.85)	17.87 (26.79)			7.68 (22.47)		12.11 (22.78)
Regularity		0.96 (0.75)	0.92 (0.80)	0.90 (0.54)	0.97 (0.59)			0.96 (1.06)		0.98 (0.71)

^a^BG: blood glucose.

^b^CGM: continuous glucose monitoring.

**Figure 1:**
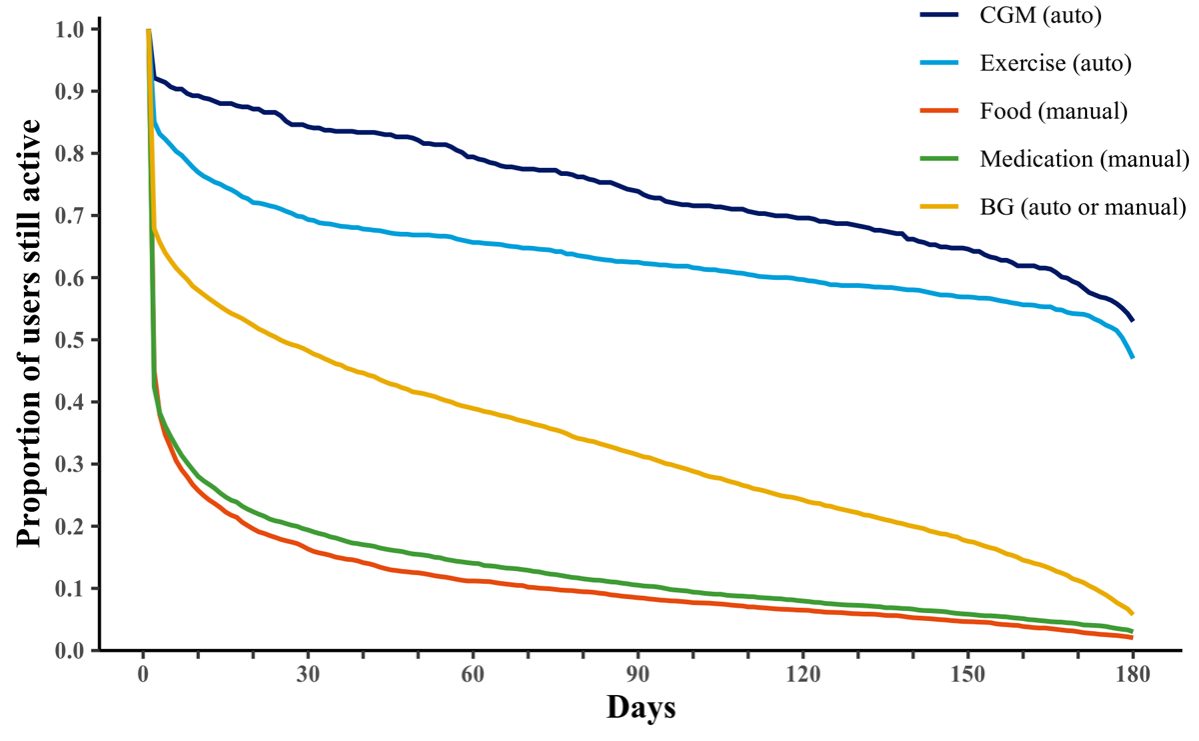
Survival curves visualizing the relative dropout of users per module. Active users are defined as active on that day or on a later day. Day 1 marks the day of initial interaction with each module. BG: blood glucose; CGM: continuous glucose monitoring.

**Figure 2:**
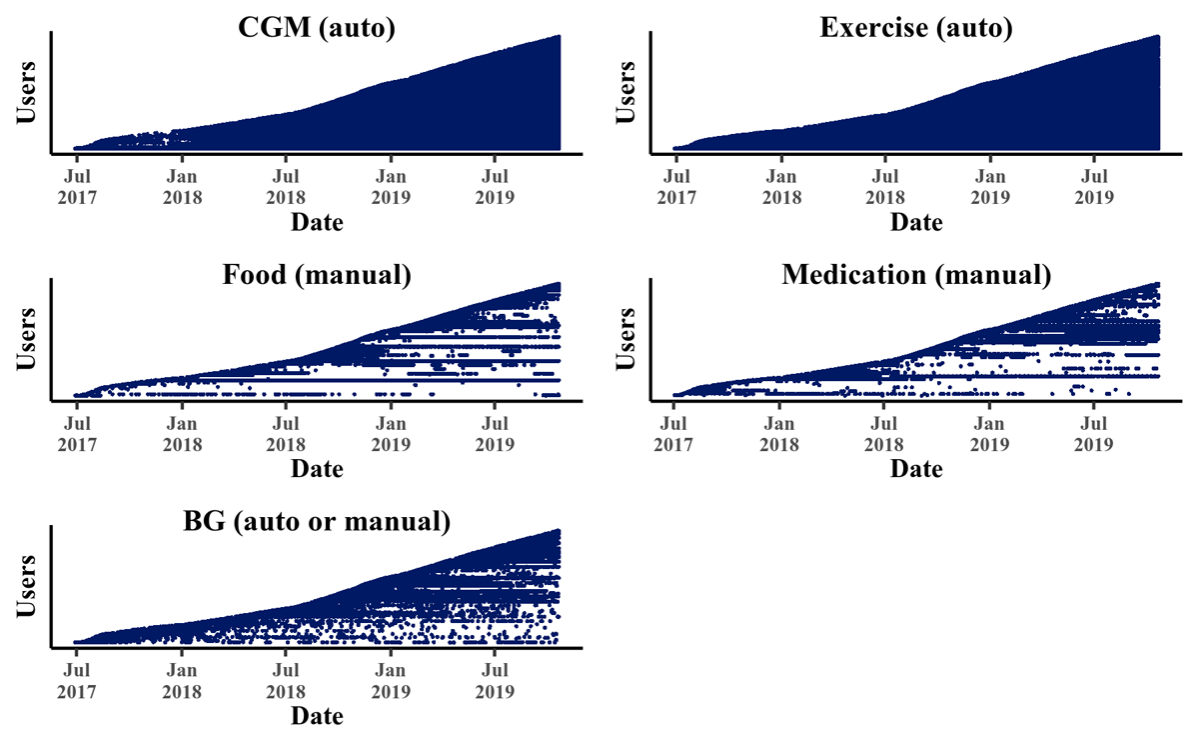
Event diagrams expressing the user activity patterns over time for the 5 modules. A dotted horizontal line represents a user’s interaction with the app, and each dot represents an active day. Dots from multiple users and days may be overlapping; however, the density of points shows a pattern of intensity. BG: blood glucose; CGM: continuous glucose monitoring.

Figures 1 and 2 support the findings in Table 3. Figure 1 emphasizes that dropout occurred faster for the modules in which use was initiated most often (food, medication, and BG). A large fraction of users dropped out already within the first week, and in the following weeks, the dropout continues to be higher than that for the other 2 modules. In contrast, more than half of the users remained active in the CGM and the exercise modules during the entire 180 days period.

Figure 2 shows the event diagrams visualizing the engagement patterns. Each dotted horizontal line represents a user’s interaction with the respective app module. The density of the graph consequently reflects all users’ activity within a module. This means that the darker the area under the curve, the more users engaged with the module. Again, the CGM and the exercise modules appear to be used most persistently, whereas the usage of the other 3 modules was often discontinued, as reflected by the white spaces.

**Figure 3:**
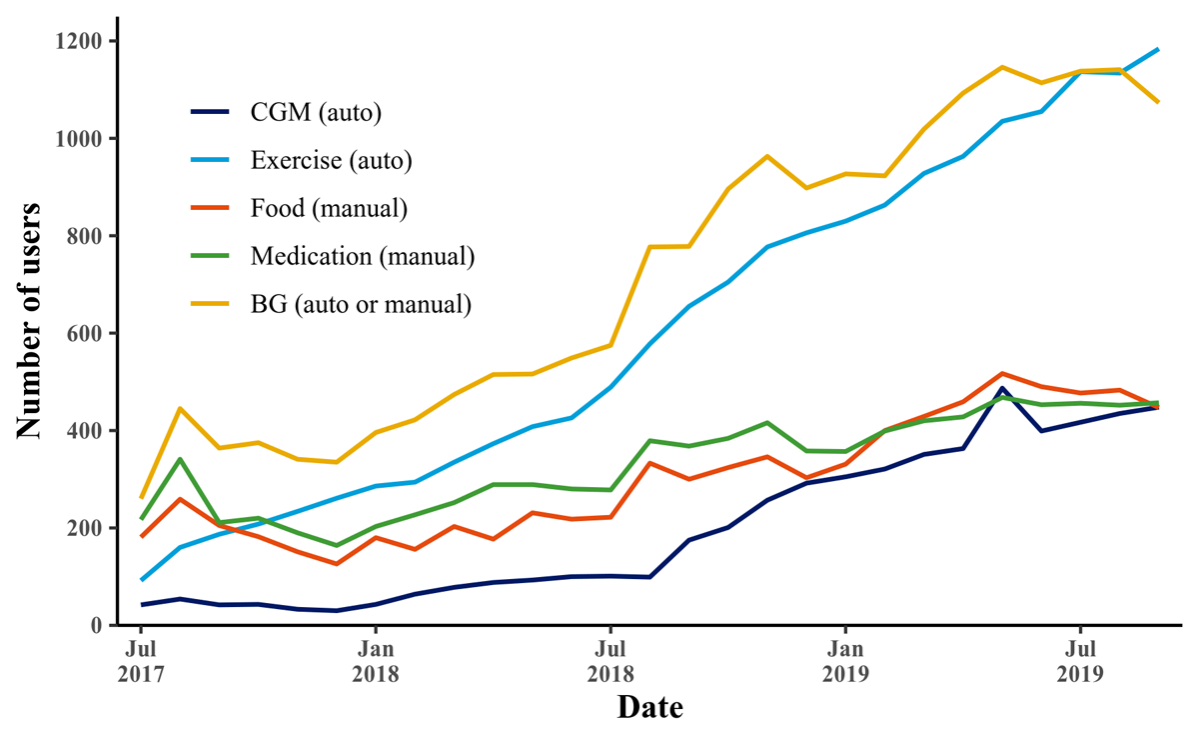
Number of active users per month. The increasing trend over time shows that more users are enrolling than dropping out. BG: blood glucose; CGM: continuous glucose monitoring.

Finally, Figure 3 adds information by showing module uptake over time. The general upward trends of the curves indicate that more individuals initiate use than drop out over time, which may indicate an increasing demand for mHealth and self-management of diabetes, for example, through successful marketing activities or other reasons for increasing awareness.

### Regression Analysis

Tables 4 and 5 summarize the results of the regression analysis, linking user characteristics and user engagement (UAR). Each column represents one module. For easier interpretation, margins are reported for each parameter and translated into active days.

Although more women used the app (2075/3649, 56.86%), the regression results indicate that they engaged significantly less with the app in 4 of the 5 modules. Only the exercise module was used significantly more by female users. Regression results further show that older users engaged significantly more with the app in all modules except the CGM module. In addition, there is a small tendency that individuals with a more recent first diabetes diagnosis used the app more intensively. The total number of actively used modules was positively associated with the interaction in a single module. Finally, body measure and glycemic control at baseline did not correlate significantly with user intensity in any module. Findings relating to diabetes type and severity of the disease do not point to a clear direction in our analysis.

To confirm robustness, the analysis was repeated with an UAR based on active weeks rather than active days. The results supported the abovementioned findings. When comparing the average number of active days and weeks in each module, it can be concluded that users do not engage with the app on concurrent days but instead interact after breaks of several days.

Applying alternative regression specifications also confirmed the robustness of our findings.

**Table 4:** Beta regressions estimating the effect of user characteristics on user engagement (user activity ratio) for nonautomated modules.

User characteristics			App module											
			Medication (n=3933; average UAR^a^=9 days)							Food (n=3821; average UAR=9 days)				
			ME^b^		*P* value		Days		ME		*P* value		Days	
**Sociodemographic**														
	**Gender (reference=male)**													
		Female		−0.019		<.001		−3.42		−0.014		<.001		−2.52
		Unknown		−0.020		<.001		−3.60		−0.015		<.001		−2.70
	**Age (reference=30-40 years)**													
		<30 years		0.000		.99		0.00		0.000		.59		0.00
		40-50 years		0.003		.23		0.54		0.003		.06		0.54
		50-60 years		0.007		.004		1.26		0.007		<.001		1.26
		60-70 years		0.009		.003		1.62		0.010		<.001		1.80
		>70 years		0.008		.098		1.44		0.009		.03		1.62
		Unknown		−0.003		.69		−0.54		0.008		.42		1.44
**BMI (** **reference** **=normal)**														
	Underweight (BMI<18.5)			−0.007		.73		−1.26		−0.020		.007		−3.60
	Overweight (25≤BMI<30)			0.010		.12		1.80		0.009		.19		1.62
	Obese (30≤BMI)			0.003		.50		0.54		−0.001		.81		−0.18
	Unknown			0.000		.99		0.00		−0.003		.50		−0.54
**Diagnosis**														
	**Type (reference=type 1 diabetes)**													
		Type 2 diabetes		0.004		.095		0.72		0.000		.89		0.00
		Prediabetes		0.016		.097		2.88		0.007		.10		1.26
		Other		0.018		.05		3.24		0.003		.48		0.54
		Unknown		0.038		<.001		6.84		0.014		.01		2.52
	**Recently diagnosed (reference=no)**													
		Yes		0.002		.16		0.36		0.003		.09		0.54
		Unknown		−0.012		.16		−2.16		−0.003		.46		−0.54
**Disease status**														
	**Treatment (reference=noninsulin)**													
		Insulin treatment		0.002		.17		0.36		−0.004		.02		−0.72
		Unknown		0.014		.12		2.52		0.007		.02		1.26
	**Blood glucose in control (reference=no)**													
		Yes		−0.005		.20		−0.90		0.000		.97		0.00
		Unknown		−0.015		<.001		−2.70		−0.012		<.001		−2.16
**Usage**														
	Number of modules used			0.015		<.001		2.70		0.009		<.001		1.62

^a^UAR: user activity ratio.

^b^ME: marginal effect reflecting the change in the outcome as the continuous variable changes by one unit (from 0 to 1 for categorical variables).

**Table 5:** Beta regressions estimating the effect of user characteristics on user engagement (user activity ratio) for (partly) automated modules.

User characteristics			App module																	
			Exercise (n=1579; average UAR^a^=67 days)						BG^b^ readings (n=5015; average UAR=13 days)							CGM^c^ (n=559; average UAR=99 days)				
			ME^d^	*P* value			Days	ME		*P* value		Days			ME		*P* value		Days	
**Sociodemographic**																				
	**Gender (reference=male)**																			
		Female	0.059		.003	10.62		−0.019			<.001		−3.42	−0.082				.02		−14.76
		Unknown	0.006		.80	10.80		−0.024			<.001		−4.32	−0.058				.15		−10.44
	**Age (reference=30-40 years)**																			
		Below 30 years	−0.067		.009	−12.06		0.002			.54		0.36	−0.026				.58		−4.68
		40-50 years	−0.008		.68	−1.44		0.007			.004		1.26	0.035				.39		6.30
		50-60 years	0.004		.85	0.72		0.015			<.001		2.70	0.022				.57		3.96
		60-70 years	0.040		.09	7.20		0.018			<.001		3.24	0.008				.87		1.44
		Above 70 years	0.032		.39	5.76		0.033			<.001		5.94	0.090				.14		16.20
		Unknown	−0.078		.37	−14.04		0.002			.77		0.36	0.068				.15		12.24
**BMI (reference=normal)**																				
	Underweight (BMI<18.5)		0.180	.64			32.4	−0.021		.30		−3.78			0.081		.54		14.58	
	Overweight (25≤BMI<30)		0.019	.63			3.42	−0.002		.72		−0.36			0.039		.40		7.02	
	Obese (30≤BMI)		0.003	.93			0.54	−0.004		.53		−0.72			0.007		.88		1.26	
	Unknown		−0.002	.94			−0.36	−0.008		.20		−1.44			0.050		.29		9.00	
**Diagnosis**																				
	**Type (reference=type 1 diabetes)**																			
		Type 2 diabetes	−0.006		.80	−1.08		0.003			.28		0.54	−0.024				.55		−4.32
		Prediabetes	−0.033		.36	−5.94		−0.003			.61		−0.54	−0.216				.12		−38.88
		Other	−0.038		.31	−6.84		0.006			.27		1.08	−0.071				.63		−12.78
		Unknown	0.022		.55	3.96		0.046			<.001		8.28	0.058				.62		10.44
	**Recently diagnosed (reference=no)**																			
		Yes	−0.021		.22	−3.78		0.005			.006		0.90	0.059				.045		11.34
		Unknown	0.013		.75	2.34		0.005			.42		0.90	0.204				.17		36.72
**Disease status**																				
	**Treatment (reference=noninsulin)**																			
		Insulin treatment	−0.005		.76	−0.90		−0.006			.002		−1.08	0.100				.13		18.00
		Unknown	−0.035		.27	−6.30		0.000			.98		0.00	−0.167				.14		−30.06
	**BG in control (reference=no)**																			
		Yes	−0.014		.49	−2.52		0.001			.43		0.18	−0.029				.61		−5.22
		Unknown	−0.045		.02	−8.10		−0.031			<.001		−5.58	−0.036				.31		−6.48
**Usage**																				
	Number of modules used		0.029	<.001			5.22	0.015		<.001		2.70			−0.002		.88		−0.36	

^a^UAR: user activity ratio.

^b^BG: blood glucose.

^c^CGM: continuous glucose monitoring.

^d^ME: marginal effect reflecting the change in the outcome as the continuous variable changes by one unit (from 0 to 1 for categorical variables).

## Discussion

### Principal Findings

The aims of this study were to investigate how intensively users engage with mHealth for diabetes and to identify patient characteristics that are associated with user engagement. Our study revealed that 42.79% (12,685/29,643) of individuals who gave consent initiated use, thus reflecting effective transition from download to use. This could reflect a superficial exploration of several apps or a general insecurity on whether and how to best integrate mHealth apps into disease self-management. The superficial exploration is likely reinforced by the large availability of different apps in the diabetes field. Furthermore, the use may be impacted by the price of the app (the app used in this study is free of charge), and if the app is selected in collaboration with an HCP [27,28]. A survey among patients with diabetes in China revealed that only 19% of the apps were recommended by HCPs, whereas most users selected their app randomly or as recommended by other patients [28]. Finally, users may be reluctant to share information due to privacy concerns. It was found that a large proportion of users did not fill out optional fields in the self-reported user profiles or entered implausible values.

In contrast to the National Diabetes Statistics Report 2020, according to which men had a higher prevalence of diagnosed diabetes, 56.86% (2075/3649) of users reported to be female [29]. This could either indicate that women are more likely to use the app or that women are more willing to share information on their gender. A total of 13.48% (1220/9051) of the users were diagnosed with T1D. Estimates from the ADA suggest that only about 5% of people with diabetes are diagnosed with T1D [30]. Explanations for the high share of users with T1D in our study may either be their higher need for constant self-monitoring (and thus demand for the app) or their usually younger age of disease onset.

For the users who initiated use, the activity delay was much higher on average for the CGM module than for the other modules. This could be explained by the increasing availability of CGM technology during the period when data for this study were collected. Therefore, some users may have gained access to a CGM device months after they consented to the app. In addition, effort and difficulty of use have been identified as the main reasons for dissatisfaction among mHealth users in a previous study [31]. Hence, the activity delay may reflect the burden of initiating the use of the CGM module, for example, due to the necessity to connect the app with an external medical device.

The average 180 days UAR varied between 0.05 and 0.55, depending on the module of the app. Comparing these numbers with existing studies is difficult because engagement metrics and reporting remain heterogeneous in the literature. However, a discrepancy between initial uptake and long-term use was found among the modules: we observed either a fast and high uptake with a lower long-term use or a lower and later uptake and use that is more continuous. Low or decreasing engagement has also been reported in previous studies among patients with diabetes (it should be noted that high churn rates have also been observed for apps in other categories and are not health or disease-specific) [13,17,19,20,32-37]. A recent study using an in-app embedded questionnaire revealed that the most satisfying user experiences took place within the first week of engagement and were related to visual elements and the feasibility of health monitoring [31]. Thus, it is a well-known challenge to keep the user engaged over time.

One reason for the dropouts in this study may be suboptimal service matching, that is, the users engage with the app but experience that the app may not fit their exact needs [38]. The C4C app offers modules targeting different aspects of diabetes treatment. The app could thus demonstrate to patients how dosing along with exercise and diet affects their diabetes. However, most users only take advantage of one specific module, which may suggest that user needs are not the same and that most users may not need the full set of functionalities. Moreover, the UAR was highest for the CGM module, that is, a new technology with potentially fewer app suppliers (ie, alternative products) on the market.

Another cause for low long-term use may be that the app is not used optimally and may therefore result in a suboptimal user experience, for example, due to missing awareness about the importance of (true) entries for effective disease management. Therefore, a rewarding experience may be crucial to keep the users who are less aware of the importance of disease monitoring engaged in the long term [31]. In contrast, it may as well be possible that a patient is reassured about his disease management after having used the app for a while and does not *need* it anymore.

In this study, the discrepancy between high uptake and fast discontinuation was particularly noticeable in the BG module, in the medication module, and in the food module. In this context, it should be emphasized that the mode of data collection varies between modules. The exercise and CGM modules automatically collect data once the app is connected to an external fitness app or the CGM device. Therefore, exploiting the module does not require any further activity from the users than opening the app. The passive data collection may thus explain the high UAR for these modules. In contrast, users must actively make entries in the medication, food, and BG modules (the number of tabs can vary depending on the exact way they choose), that is, modules with lower engagement. Accordingly, the median number of seconds to perform an entry was lowest in the CGM module (0 seconds) and in the exercise module (0 seconds, if from a connected app), followed by the BG module (58 seconds), the medication module (117 seconds), and the food module (156 seconds; Multimedia Appendix 1). Therefore, the mode of data collection may be an important driver for engagement with the app. This finding is in accordance with the existing literature based on surveys. The studies revealed that the data entry burden is the main reason for discontinuation of use, and the authors suggest that enabling features with automatically transmitted data would increase the users’ compliance [27,28]. However, it should be pointed out that it remains unclear how actively users ultimately interact with the app when data have been transmitted automatically. As the app needs to be opened to sync, the engagement metrics can (at least) serve as a proxy for user engagement. Furthermore, besides engagement, the metrics inform about the amount of data collected and reflect the availability of rich health information. Previous studies showed that both patients and diabetologists thought that diaries and doctor-patient communication based on collected data were the most important features of apps for diabetes [28,39]. On the other hand, we found that the medication, food, and BG modules had a higher proportion of users than the other modules, potentially because the manual data entry mode makes it possible for any person with diabetes to register data (for the BG module, a BG meter is necessary, but these are very common for people with diabetes). In contrast, the CGM and exercise modules are meant for synchronization with devices or apps, which the user may not have or which pose an additional burden and complexity to initiate use.

Our regression results indicate that the optimal approach to increase user engagement may be patient specific and depend on their individual characteristics. In line with the findings of Rahman et al [18], we concluded that although most users of the app were female, male users were significantly more likely to be engaged in the app. Only the exercise module was used significantly more by female users. This result could be explained by a higher body consciousness, which is in accordance with existing literature revealing that women reported higher exercise levels than men [40]. Another explanation may be that exercise data are often passively collected and would require active disconnection from the external app. Furthermore, we showed that older users engaged significantly more with the app. Compared with previous studies, this finding is rather novel. On the basis of the interviews with patients aged 50 years or older, Scheibe et al [41] revealed a lack of acceptance and a low use of diabetes apps among the elderly, mainly due to a lack of additional benefits and ease of operation. Similarly, using survey data, Zhang et al [28] showed that the use of diabetes apps decreased with patient age. One explanation for our finding may be that if older individuals overcome a potential lack of knowledge in app use, they may be motivated to apply their newly obtained skills. In contrast, younger mobile device owners might consider their phone as a spare time activity and have a lower burden downloading and trying different apps [42]. Moreover, considering that diabetes is a chronic condition, the older may be more severely ill, and therefore, they require more (self-) monitoring compared with younger patients in earlier stages of the disease.

In addition, our analysis reveals that there is a tendency that individuals with a more recent first diabetes diagnosis used the app more intensively. So far, the existing literature could not find a correlation between disease duration and app use [28]. However, it is possible that new patients have considered their mobile phone as a helpful tool when establishing their initial individual self-management strategy. The great potential of patient support apps is increasingly emphasized by official institutions, such as the ADA or the WHO [11,43]. In contrast, long-term patients may not consider adjusting their predeveloped disease management habits. A usability study with older adults showed that participants were already satisfied with their current management system and that they would need to find a reason why apps are superior to their current medication management system [44]. However, it should be noted that our observation is only based on an approximate measure for the time of diagnosis, and more research is needed to confirm this finding.

The total number of actively used modules was positively associated with the interaction in a single module. This could indicate synergy effects among the modules, if the app was integrated in managing more aspects of a user’s disease or could reflect a higher discipline of users who take advantage of multiple modules.

### Limitations

Our study has several limitations. First, missing data is a common problem when analyzing self-reported real-world data, ranging from 9.88% (894/9051) of users who did not share information about their diabetes type to 63.31% (5730/9051) of users who did not share information about their height or weight. We addressed this by categorizing missing data as unknown. In the context of this study, the amount of missing data can be viewed as an important result in itself. It is noteworthy that many unknown categories are highly significant. This may indicate that there are factors associated with user engagement that could not be observed in the given data set. Second, in a broader sense, data limitations constrained our ability to investigate other important factors that may influence user engagement, such as education or patient-doctor interactions. Wherever possible, we generated proxies substituting unobserved variables of interest. Finally, the data set did not cover information about the log-in times. Instead, our study was solely based on data inputs. Although we emphasized the relevance of automated data collection in our discussion, it remains unclear how actively engaged the users actually are, because collecting data is not the same as using data. In this context, it should be the ultimate goal of future research to assess how engagement with mHealth affects health outcomes and which module is most important from the health perspective.

### Conclusions

There is no consensus on how to assess user engagement with mHealth solutions, but it is needed. This paper proposes a set of theoretically founded user engagement metrics that were used to assess user engagement in this study. After providing consent, 42.79% (12,685/29,643) of the users initiated use. Most users took advantage of one specific module of the app, indicating that the needs of patients with diabetes are highly heterogeneous. User engagement and amount of collected data were higher for automated modules, although initial uptake remained lower for these modules. Therefore, to increase the use of apps, providers of mHealth should consider the mode of data gathering and content design and take into account the privacy concerns of the users at the same time. Users’ engagement was determined by various patient characteristics. Although most users reported to be female, male users engaged significantly more with the app. Older people and users who were recently diagnosed tended to use the app more actively. This indicates that particular patient groups should be specifically targeted or assisted when integrating apps into the self-management of their disease.

## Appendix

Multimedia Appendix 1 Users’ burden to do an entry in each module.

## Abbreviations

ADA: American Diabetes Association
BG: blood glucose
C4C: Cornerstones4Care
CGM: continuous glucose monitoring
HCP: health care professional
mHealth: mobile health
T1D: type 1 diabetes
T2D: type 2 diabetes
TIR: time in range
UAR: user activity ratio
WHO: World Health Organization
