# Supplementary material for: Real-World Evidence of User Engagement With Mobile Health for Diabetes Management: Longitudinal Observational Study
Source: JMIR Mhealth Uhealth. 2020 Nov 6;8(11):e22212. doi: 10.2196/22212 (PMC7679206; doi:10.2196/22212)
Supplement: Multimedia Appendix 1 [file mhealth_v8i11e22212_app1.docx]

## Multimedia Appendix: Users’ burden to do an entry in each module

This is a Multimedia Appendix to a full manuscript published in the J Med Internet Res. For full copyright and citation information, see http://dx.doi.org/10.2196/jmir.22212.

Table A 1: Users’ burden to do an entry in each module

| Module | Minimum no. of clicks to do an entry | Median no. of seconds  to do an entry |
| --- | --- | --- |
| BG | If new device (never synced) - 8 taps  If existing device (previously synced) - 3 taps | 58 |
|  |  |  |
| Medication | If via Recents - 6 taps  If via Medication database - 10 taps | 117 |
|  |  |  |
| Food | If via Recents - 4 taps  If via food database - 8 taps  If via barcode scanner - 5 taps | 156 |
|  |  |  |
| Manual exercise | If via Recents - 4 taps If via Exercise database - 8 taps | 186 |
|  |  |  |
| Exercise from connected app | 8-12 tap initially (varies by device) | -- |
|  |  |  |
| CGM | 8-12 tap initially (varies by device) | -- |
